# 2,4-Dibromo-6-[(*E*)-({3-[(*E*)-(3,5-dibromo-2-oxidobenzyl­idene)aza­nium­yl]-2,2-dimethyl­prop­yl}iminium­yl)meth­yl]phenolate

**DOI:** 10.1107/S1600536811055899

**Published:** 2012-01-11

**Authors:** Hadi Kargar, Reza Kia, Mahbubeh Haghshenas, Muhammad Nawaz Tahir

**Affiliations:** a Department of Chemistry, Payame Noor University, PO BOX 19395-3697 Tehran, I. R. of IRAN; b X-ray Crystallography Lab., Plasma Physics Research Center, Science and Research Branch, Islamic Azad University, Tehran, Iran; c Department of Chemistry, Science and Research Branch, Islamic Azad University, Tehran, Iran; d Department of Physics, University of Sargodha, Punjab, Pakistan

## Abstract

In the title mol­ecule, C_19_H_18_Br_4_N_2_O_2_, the dihedral angle between the benzene rings is 73.9 (2)°. Two intra­molecular N—H⋯O hydrogen bonds make *S*(6) ring motifs. In the crystal, mol­ecules are linked *via* C—H⋯O inter­actions, forming chains propagating along the *a*-axis directon. A short C⋯Br [3.401 (5) Å] contact is present in the crystal structure, which is further stabilized by a π–π inter­action [centroid–centroid distance = 3.739 (3) Å].

## Related literature

For standard bond lengths, see: Allen *et al.* (1987 ▶). For hydrogen bond motifs, see: Bernstein *et al.* (1995 ▶). For related structures, see: Kargar *et al.* (2011 ▶); Kia *et al.* (2010 ▶).
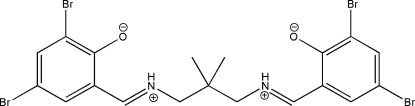


## Experimental

### 

#### Crystal data

C_19_H_18_Br_4_N_2_O
*M* *_r_* = 625.99
Orthorhombic, 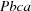

*a* = 11.6861 (3) Å
*b* = 11.4616 (3) Å
*c* = 31.3782 (9) Å
*V* = 4202.8 (2) Å^3^
*Z* = 8
Mo *K*α radiation
μ = 7.68 mm^−1^
*T* = 291 K
0.25 × 0.16 × 0.12 mm

#### Data collection

Bruker SMART APEXII CCD area-detector diffractometer
Absorption correction: multi-scan (*SADABS*; Bruker, 2005 ▶) *T*_min_ = 0.250, *T*_max_ = 0.459
38547 measured reflections
5242 independent reflections
2756 reflections with *I* > 2σ(*I*)
*R*_int_ = 0.090

#### Refinement

*R*[*F* ^2^ > 2σ(*F* ^2^)] = 0.047
*wR*(*F* ^2^) = 0.138
*S* = 1.04
5242 reflections
246 parameters
H-atom parameters constrained
Δρ_max_ = 0.73 e Å^−3^
Δρ_min_ = −0.64 e Å^−3^

### 

Data collection: *APEX2* (Bruker, 2005 ▶); cell refinement: *SAINT* (Bruker, 2005 ▶); data reduction: *SAINT*; program(s) used to solve structure: *SHELXS97* (Sheldrick, 2008 ▶); program(s) used to refine structure: *SHELXL97* (Sheldrick, 2008 ▶); molecular graphics: *SHELXTL* (Sheldrick, 2008 ▶); software used to prepare material for publication: *SHELXTL* and *PLATON* (Spek, 2009 ▶).

## Supplementary Material

Crystal structure: contains datablock(s) global, I. DOI: 10.1107/S1600536811055899/su2354sup1.cif

Structure factors: contains datablock(s) I. DOI: 10.1107/S1600536811055899/su2354Isup2.hkl

Additional supplementary materials:  crystallographic information; 3D view; checkCIF report

## Figures and Tables

**Table 1:** Hydrogen-bond geometry (Å, °)

*D*—H⋯*A*	*D*—H	H⋯*A*	*D*⋯*A*	*D*—H⋯*A*
N1—H1⋯O1	0.85	1.82	2.549 (5)	142
N2—H2⋯O2	0.86	1.80	2.537 (5)	143
C8—H8*A*⋯O2^i^	0.97	2.53	3.424 (7)	152

Symmetry code: (i) 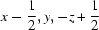.

**Fig. 1.**
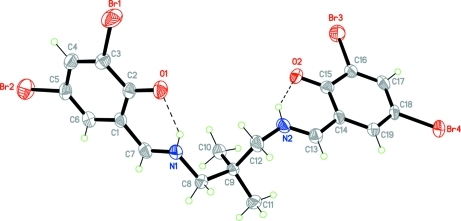
The molecular structure of the title molecule, showing 40% probability displacement ellipsoids and the atomic numbering. The dashed lines show the intramolecular N-H···O hydrogen bonds.

**Fig. 2.**
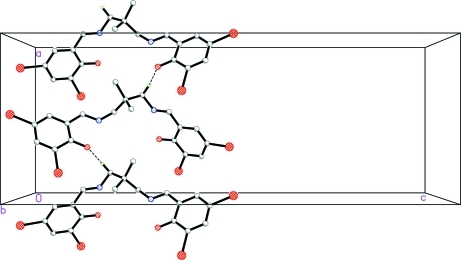
The crystal packing of the title compound, viewed along the b-axis, showing linking of molecules into a chain along the a-axis through the intermolecular C—H···O intearctions (dashed lines). Only the H atoms involved in these interactions are shown.

## supplementary crystallographic information

### Comment

In continuation of our work on the crystal structure analysis of Schiff base ligands (Kargar *et al.*, 2011; Kia *et al.*, 2010), we synthesized the title compound and report herein on its crystal structure.

The title compound (Fig. 1) is a potential tetradentate Zwitterionic Schiff base ligand. The bond lengths (Allen *et al.*, 1987) and angles are within normal ranges and are comparable to thoses observed for related structures (Kargar *et al.*, 2011; Kia *et al.*, 2010).

In the molecule there are two intramolecular N—H···O hydrogen bonds (Table 1) making S(6) ring motifs (Bernstein *et al.*, 1995). The dihedral angle between the benzene rings is 73.9 (2)°. An interesting feature of the crystal structure is the short C6···Br1^ii^ contact [3.401 (5) Å; (ii) -*x+1/2*, *y-1/2*, *z*] , which is shorter than the sum of the van der Waals radii [3.55 Å] of these atoms.

In the crystal, molecules are linked together along the *a* axis into chains through an intermolecular C—H···O interaction (Fig. 2 and Table 1). The crystal structure is further stabilized by an intermolecular π-π interaction [*Cg*1···*Cg*2^iii^ = 3.739 (3)Å; (iii) x, -y*-1/2*, z*+1/2*; *Cg*1 and *Cg*2 are the centroid of benzene rings (C1–C6) and (C14–19), respectively].

### Experimental

The title compound was synthesized by adding 3,5-dibromo-salicylaldehyde (2 mmol) to a solution of 2,2-dimethyl-1,3-propanediamine (1 mmol) in ethanol (30 ml). The mixture was refluxed with stirring for 30 min. The resultant solution was filtered. Yellow single crystals of the title compound, suitable for *X*-ray structure determination, were recrystallized from ethanol by slow evaporation of the solvent at room temperature over several days.

### Refinement

The NH H-atoms were located in a difference Fourier map and were refined as riding atoms with U_iso_ (H) = 1.2 U_eq_ (N). The C-bound H-atoms were included in calculated positions and treated as riding atoms: C-H = 0.93, 0.97 and 0.96 Å for CH, CH_2_, and CH_3_ H-atoms, respectively, with U_iso_(H) = k × U_eq_(parent C-atom), where k = 1.5 for CH_3_ H-atoms and k = 1.2 for all other H-atoms.

### Crystal data

**Table:**

C_19_H_18_Br_4_N_2_O	*F*(000) = 2416
*M**_r_* = 625.99	*D*_x_ = 1.979 Mg m^−^^3^
Orthorhombic, *P**b**c**a*	Mo *K*α radiation, λ = 0.71073 Å
Hall symbol: -P 2ac 2ab	Cell parameters from 2370 reflections
*a* = 11.6861 (3) Å	θ = 2.5–27.5°
*b* = 11.4616 (3) Å	µ = 7.68 mm^−^^1^
*c* = 31.3782 (9) Å	*T* = 291 K
*V* = 4202.8 (2) Å^3^	Block, yellow
*Z* = 8	0.25 × 0.16 × 0.12 mm

### Data collection

**Table:**

Bruker SMART APEXII CCD area-detector diffractometer	5242 independent reflections
Radiation source: fine-focus sealed tube	2756 reflections with *I* > 2σ(*I*)
graphite	*R*_int_ = 0.090
φ and ω scans	θ_max_ = 28.4°, θ_min_ = 1.3°
Absorption correction: multi-scan (*SADABS*; Bruker, 2005)	*h* = −15→15
*T*_min_ = 0.250, *T*_max_ = 0.459	*k* = −15→15
38547 measured reflections	*l* = −41→41

### Refinement

**Table:**

Refinement on *F*^2^	Primary atom site location: structure-invariant direct methods
Least-squares matrix: full	Secondary atom site location: difference Fourier map
*R*[*F*^2^ > 2σ(*F*^2^)] = 0.047	Hydrogen site location: inferred from neighbouring sites
*wR*(*F*^2^) = 0.138	H-atom parameters constrained
*S* = 1.04	*w* = 1/[σ^2^(*F*_o_^2^) + (0.0616*P*)^2^] where *P* = (*F*_o_^2^ + 2*F*_c_^2^)/3
5242 reflections	(Δ/σ)_max_ = 0.001
246 parameters	Δρ_max_ = 0.73 e Å^−^^3^
0 restraints	Δρ_min_ = −0.64 e Å^−^^3^

### Special details

**Table:**

Geometry. All esds (except the esd in the dihedral angle between two l.s. planes) are estimated using the full covariance matrix. The cell esds are taken into account individually in the estimation of esds in distances, angles and torsion angles; correlations between esds in cell parameters are only used when they are defined by crystal symmetry. An approximate (isotropic) treatment of cell esds is used for estimating esds involving l.s. planes.
Refinement. Refinement of F^2^ against ALL reflections. The weighted R-factor wR and goodness of fit S are based on F^2^, conventional R-factors R are based on F, with F set to zero for negative F^2^. The threshold expression of F^2^ > 2sigma(F^2^) is used only for calculating R-factors(gt) etc. and is not relevant to the choice of reflections for refinement. R-factors based on F^2^ are statistically about twice as large as those based on F, and R- factors based on ALL data will be even larger.

### Fractional atomic coordinates and isotropic or equivalent isotropic displacement parameters (Å^2^)

**Table:**

	*x*	*y*	*z*	*U*_iso_*/*U*_eq_	
Br1	0.34182 (5)	−0.30064 (6)	0.37158 (2)	0.0653 (2)	
Br2	0.17302 (6)	0.00225 (6)	0.49759 (2)	0.0695 (2)	
Br3	0.32582 (5)	0.27233 (8)	0.11028 (2)	0.0748 (3)	
Br4	−0.02936 (5)	0.17726 (6)	−0.006978 (18)	0.0531 (2)	
O1	0.1272 (3)	−0.2212 (3)	0.32548 (12)	0.0456 (9)	
O2	0.1731 (3)	0.1290 (3)	0.16822 (12)	0.0493 (10)	
N1	−0.0616 (3)	−0.1131 (4)	0.31559 (14)	0.0399 (11)	
H1	−0.0092	−0.1601	0.3081	0.048*	
N2	0.0054 (3)	−0.0002 (3)	0.18846 (14)	0.0401 (11)	
H2	0.0681	0.0364	0.1930	0.048*	
C1	0.0493 (4)	−0.0982 (4)	0.37894 (16)	0.0339 (11)	
C2	0.1341 (4)	−0.1743 (4)	0.36298 (16)	0.0348 (12)	
C3	0.2273 (4)	−0.1966 (4)	0.39035 (17)	0.0399 (13)	
C4	0.2376 (4)	−0.1472 (5)	0.42992 (17)	0.0430 (13)	
H4	0.3002	−0.1645	0.4471	0.052*	
C5	0.1525 (4)	−0.0701 (5)	0.44416 (17)	0.0417 (13)	
C6	0.0597 (4)	−0.0475 (4)	0.41923 (17)	0.0398 (13)	
H6	0.0026	0.0022	0.4291	0.048*	
C7	−0.0502 (4)	−0.0723 (5)	0.35284 (17)	0.0396 (13)	
H7	−0.1072	−0.0243	0.3638	0.047*	
C8	−0.1584 (4)	−0.0846 (5)	0.28844 (17)	0.0422 (13)	
H8A	−0.2174	−0.0478	0.3055	0.051*	
H8B	−0.1899	−0.1560	0.2767	0.051*	
C9	−0.1256 (4)	−0.0031 (4)	0.25192 (16)	0.0351 (12)	
C10	−0.0651 (4)	0.1058 (4)	0.26898 (17)	0.0438 (13)	
H10A	−0.1097	0.1392	0.2916	0.066*	
H10B	−0.0568	0.1618	0.2464	0.066*	
H10C	0.0090	0.0848	0.2796	0.066*	
C11	−0.2360 (4)	0.0326 (5)	0.22920 (19)	0.0528 (16)	
H11A	−0.2745	−0.0359	0.2189	0.079*	
H11B	−0.2180	0.0827	0.2056	0.079*	
H11C	−0.2849	0.0732	0.2488	0.079*	
C12	−0.0487 (4)	−0.0721 (5)	0.22116 (17)	0.0444 (13)	
H12A	0.0105	−0.1111	0.2375	0.053*	
H12B	−0.0943	−0.1318	0.2073	0.053*	
C13	−0.0332 (4)	0.0110 (4)	0.15044 (17)	0.0379 (12)	
H13	−0.1005	−0.0271	0.1429	0.045*	
C14	0.0254 (4)	0.0815 (4)	0.11903 (15)	0.0338 (11)	
C15	0.1304 (4)	0.1368 (4)	0.13050 (17)	0.0365 (12)	
C16	0.1851 (4)	0.2010 (5)	0.09768 (18)	0.0407 (13)	
C17	0.1389 (4)	0.2107 (4)	0.05729 (17)	0.0412 (13)	
H17	0.1776	0.2522	0.0363	0.049*	
C18	0.0336 (4)	0.1579 (4)	0.04800 (15)	0.0354 (11)	
C19	−0.0218 (4)	0.0939 (4)	0.07783 (16)	0.0365 (12)	
H19	−0.0909	0.0580	0.0712	0.044*	

### Atomic displacement parameters (Å^2^)

**Table:**

	*U*^11^	*U*^22^	*U*^33^	*U*^12^	*U*^13^	*U*^23^
Br1	0.0620 (4)	0.0651 (4)	0.0688 (5)	0.0276 (3)	−0.0133 (3)	−0.0081 (4)
Br2	0.0780 (5)	0.0835 (5)	0.0470 (4)	−0.0080 (4)	−0.0106 (3)	−0.0238 (3)
Br3	0.0533 (4)	0.1174 (6)	0.0537 (4)	−0.0359 (4)	0.0020 (3)	0.0015 (4)
Br4	0.0591 (4)	0.0723 (4)	0.0279 (3)	0.0027 (3)	−0.0031 (3)	0.0027 (3)
O1	0.056 (2)	0.048 (2)	0.033 (2)	0.0070 (18)	−0.0035 (18)	−0.0054 (18)
O2	0.043 (2)	0.071 (3)	0.034 (2)	−0.0015 (18)	−0.0082 (18)	0.008 (2)
N1	0.037 (2)	0.051 (3)	0.032 (3)	0.0050 (19)	0.001 (2)	0.005 (2)
N2	0.040 (2)	0.046 (3)	0.035 (3)	0.0020 (19)	0.007 (2)	0.008 (2)
C1	0.039 (3)	0.034 (3)	0.029 (3)	−0.001 (2)	0.002 (2)	0.007 (2)
C2	0.044 (3)	0.030 (3)	0.031 (3)	−0.004 (2)	−0.002 (2)	0.008 (2)
C3	0.042 (3)	0.033 (3)	0.044 (4)	−0.001 (2)	−0.005 (3)	0.007 (3)
C4	0.043 (3)	0.048 (3)	0.038 (3)	−0.004 (3)	−0.008 (3)	0.002 (3)
C5	0.053 (3)	0.043 (3)	0.029 (3)	−0.010 (3)	−0.004 (3)	−0.001 (2)
C6	0.046 (3)	0.040 (3)	0.033 (3)	−0.001 (2)	0.004 (3)	0.001 (3)
C7	0.046 (3)	0.040 (3)	0.033 (3)	0.003 (2)	0.008 (3)	0.007 (2)
C8	0.035 (3)	0.060 (4)	0.032 (3)	−0.003 (3)	−0.005 (2)	0.010 (3)
C9	0.030 (2)	0.048 (3)	0.028 (3)	0.002 (2)	0.003 (2)	0.010 (2)
C10	0.046 (3)	0.049 (3)	0.037 (3)	−0.001 (3)	0.003 (3)	0.006 (3)
C11	0.036 (3)	0.077 (4)	0.045 (4)	−0.001 (3)	−0.005 (3)	0.021 (3)
C12	0.052 (3)	0.048 (3)	0.033 (3)	0.001 (3)	0.004 (3)	0.011 (3)
C13	0.041 (3)	0.037 (3)	0.036 (3)	−0.005 (2)	0.000 (3)	−0.001 (2)
C14	0.040 (3)	0.037 (3)	0.024 (3)	0.005 (2)	0.003 (2)	−0.005 (2)
C15	0.033 (3)	0.038 (3)	0.039 (3)	0.002 (2)	0.001 (2)	−0.004 (3)
C16	0.036 (3)	0.050 (3)	0.036 (3)	−0.006 (2)	0.005 (2)	−0.002 (3)
C17	0.046 (3)	0.044 (3)	0.033 (3)	0.003 (3)	0.012 (3)	0.005 (3)
C18	0.046 (3)	0.039 (3)	0.022 (3)	0.005 (2)	0.000 (2)	−0.005 (2)
C19	0.035 (3)	0.043 (3)	0.031 (3)	−0.001 (2)	0.000 (2)	−0.006 (2)

### Geometric parameters (Å, °)

**Table:**

Br1—C3	1.887 (5)	C8—H8A	0.9700
Br2—C5	1.886 (5)	C8—H8B	0.9700
Br3—C16	1.879 (5)	C9—C11	1.530 (7)
Br4—C18	1.889 (5)	C9—C10	1.531 (7)
O1—C2	1.296 (6)	C9—C12	1.538 (7)
O2—C15	1.287 (6)	C10—H10A	0.9600
N1—C7	1.266 (6)	C10—H10B	0.9600
N1—C8	1.453 (6)	C10—H10C	0.9600
N1—H1	0.8479	C11—H11A	0.9600
N2—C13	1.282 (6)	C11—H11B	0.9600
N2—C12	1.460 (6)	C11—H11C	0.9600
N2—H2	0.8557	C12—H12A	0.9700
C1—C6	1.397 (7)	C12—H12B	0.9700
C1—C2	1.412 (7)	C13—C14	1.447 (7)
C1—C7	1.454 (7)	C13—H13	0.9300
C2—C3	1.410 (7)	C14—C19	1.412 (7)
C3—C4	1.370 (7)	C14—C15	1.427 (7)
C4—C5	1.403 (7)	C15—C16	1.418 (7)
C4—H4	0.9300	C16—C17	1.382 (7)
C5—C6	1.363 (7)	C17—C18	1.402 (7)
C6—H6	0.9300	C17—H17	0.9300
C7—H7	0.9300	C18—C19	1.354 (7)
C8—C9	1.527 (7)	C19—H19	0.9300
			
C7—N1—C8	122.6 (5)	C9—C10—H10A	109.5
C7—N1—H1	114.5	C9—C10—H10B	109.5
C8—N1—H1	122.9	H10A—C10—H10B	109.5
C13—N2—C12	123.9 (5)	C9—C10—H10C	109.5
C13—N2—H2	114.0	H10A—C10—H10C	109.5
C12—N2—H2	122.1	H10B—C10—H10C	109.5
C6—C1—C2	121.2 (5)	C9—C11—H11A	109.5
C6—C1—C7	119.6 (5)	C9—C11—H11B	109.5
C2—C1—C7	119.2 (5)	H11A—C11—H11B	109.5
O1—C2—C3	121.8 (5)	C9—C11—H11C	109.5
O1—C2—C1	122.3 (5)	H11A—C11—H11C	109.5
C3—C2—C1	115.9 (5)	H11B—C11—H11C	109.5
C4—C3—C2	123.1 (5)	N2—C12—C9	113.8 (4)
C4—C3—Br1	118.8 (4)	N2—C12—H12A	108.8
C2—C3—Br1	118.1 (4)	C9—C12—H12A	108.8
C3—C4—C5	119.1 (5)	N2—C12—H12B	108.8
C3—C4—H4	120.4	C9—C12—H12B	108.8
C5—C4—H4	120.4	H12A—C12—H12B	107.7
C6—C5—C4	120.1 (5)	N2—C13—C14	121.5 (5)
C6—C5—Br2	121.9 (4)	N2—C13—H13	119.2
C4—C5—Br2	118.0 (4)	C14—C13—H13	119.2
C5—C6—C1	120.6 (5)	C19—C14—C15	121.5 (5)
C5—C6—H6	119.7	C19—C14—C13	119.6 (5)
C1—C6—H6	119.7	C15—C14—C13	118.9 (5)
N1—C7—C1	121.9 (5)	O2—C15—C16	121.9 (5)
N1—C7—H7	119.1	O2—C15—C14	122.3 (5)
C1—C7—H7	119.1	C16—C15—C14	115.8 (5)
N1—C8—C9	112.5 (4)	C17—C16—C15	122.1 (5)
N1—C8—H8A	109.1	C17—C16—Br3	120.0 (4)
C9—C8—H8A	109.1	C15—C16—Br3	117.9 (4)
N1—C8—H8B	109.1	C16—C17—C18	119.9 (5)
C9—C8—H8B	109.1	C16—C17—H17	120.0
H8A—C8—H8B	107.8	C18—C17—H17	120.0
C8—C9—C11	107.5 (4)	C19—C18—C17	120.7 (5)
C8—C9—C10	110.6 (4)	C19—C18—Br4	120.6 (4)
C11—C9—C10	109.6 (4)	C17—C18—Br4	118.8 (4)
C8—C9—C12	107.6 (4)	C18—C19—C14	120.0 (5)
C11—C9—C12	109.7 (4)	C18—C19—H19	120.0
C10—C9—C12	111.7 (4)	C14—C19—H19	120.0
			
C6—C1—C2—O1	−179.0 (4)	C13—N2—C12—C9	−97.7 (6)
C7—C1—C2—O1	0.9 (7)	C8—C9—C12—N2	−170.1 (4)
C6—C1—C2—C3	1.0 (7)	C11—C9—C12—N2	73.2 (5)
C7—C1—C2—C3	−179.1 (4)	C10—C9—C12—N2	−48.5 (6)
O1—C2—C3—C4	179.1 (5)	C12—N2—C13—C14	−178.6 (4)
C1—C2—C3—C4	−0.9 (7)	N2—C13—C14—C19	−178.4 (5)
O1—C2—C3—Br1	−1.6 (6)	N2—C13—C14—C15	1.9 (7)
C1—C2—C3—Br1	178.4 (3)	C19—C14—C15—O2	178.1 (5)
C2—C3—C4—C5	−0.4 (8)	C13—C14—C15—O2	−2.2 (7)
Br1—C3—C4—C5	−179.7 (4)	C19—C14—C15—C16	−1.9 (7)
C3—C4—C5—C6	1.7 (8)	C13—C14—C15—C16	177.8 (4)
C3—C4—C5—Br2	−177.0 (4)	O2—C15—C16—C17	−179.2 (5)
C4—C5—C6—C1	−1.5 (8)	C14—C15—C16—C17	0.8 (7)
Br2—C5—C6—C1	177.0 (4)	O2—C15—C16—Br3	1.7 (7)
C2—C1—C6—C5	0.2 (8)	C14—C15—C16—Br3	−178.3 (3)
C7—C1—C6—C5	−179.7 (5)	C15—C16—C17—C18	1.2 (8)
C8—N1—C7—C1	−177.6 (4)	Br3—C16—C17—C18	−179.7 (4)
C6—C1—C7—N1	177.6 (5)	C16—C17—C18—C19	−2.3 (8)
C2—C1—C7—N1	−2.2 (7)	C16—C17—C18—Br4	177.9 (4)
C7—N1—C8—C9	107.5 (6)	C17—C18—C19—C14	1.2 (7)
N1—C8—C9—C11	−173.0 (5)	Br4—C18—C19—C14	−179.0 (4)
N1—C8—C9—C10	−53.4 (6)	C15—C14—C19—C18	1.0 (7)
N1—C8—C9—C12	68.8 (6)	C13—C14—C19—C18	−178.7 (4)

### Hydrogen-bond geometry (Å, °)

**Table:**

*D*—H···*A*	*D*—H	H···*A*	*D*···*A*	*D*—H···*A*
N1—H1···O1	0.85	1.82	2.549 (5)	142
N2—H2···O2	0.86	1.80	2.537 (5)	143
C8—H8A···O2^i^	0.97	2.53	3.424 (7)	152

Symmetry codes: (i) *x*−1/2, *y*, −*z*+1/2.

## References

[bb1] Allen, F. H., Kennard, O., Watson, D. G., Brammer, L., Orpen, A. G. & Taylor, R. (1987). *J. Chem. Soc. Perkin Trans. 2*, pp. S1–19.

[bb2] Bernstein, J., Davis, R. E., Shimoni, L. & Chang, N.-L. (1995). *Angew. Chem. Int. Ed. Engl.* **34**, 1555–1573.

[bb3] Bruker (2005). *APEX2*, *SAINT* and *SADABS* Bruker AXS Inc., Madison, Wisconsin, USA.

[bb4] Kargar, H., Kia, R., Pahlavani, E. & Tahir, M. N. (2011). *Acta Cryst.* E**67**, o614. 10.1107/S1600536811004776 PMC3052006 21522371

[bb5] Kia, R., Kargar, H., Tahir, M. N. & Kianoosh, F. (2010). *Acta Cryst.* E**66**, o2296. 10.1107/S1600536810031430 PMC3008013 21588648

[bb6] Sheldrick, G. M. (2008). *Acta Cryst.* A**64**, 112–122. 10.1107/S0108767307043930 18156677

[bb7] Spek, A. L. (2009). *Acta Cryst.* D**65**, 148–155. 10.1107/S090744490804362X PMC2631630 19171970

